# Development of a rapid HPLC-fluorescence method for monitoring warfarin metabolites formation: In vitro studies for evaluating the effect of piperine on warfarin metabolism and plasma coagulation

**DOI:** 10.1016/j.heliyon.2024.e31266

**Published:** 2024-05-14

**Authors:** Aref L. Zayed, Mohammad Hadieh, Jomana Al Hroot, Fatima Hameedat, Sana'a A. Jaber

**Affiliations:** Department of Medicinal Chemistry and Pharmacognosy, Faculty of Pharmacy, Jordan University of Science and Technology, P.O. Box 3030, Irbid, 22110, Jordan

**Keywords:** Warfarin, Metabolism, Piperine, Liver microsomes, 7-Hydroxywarfarin, Prothrombin time (PT), HPLC, Fluorescence

## Abstract

Warfarin, a widely prescribed anticoagulant, is highly effective for various coagulation disorders. However, its efficacy is limited by a narrow therapeutic index and frequent drug interactions, especially those involving metabolism by Cytochrome P450 (CYP450) enzymes. Piperine, found in black and long pepper, possesses blood-thinning properties and has been observed to inhibit CYP3A and CYP2C enzymes linked to warfarin metabolism. This study investigated the effect of piperine on warfarin metabolism in liver microsomes using a rapid and sensitive HPLC-Fluorescence method. The use of PFP (pentafluorophenyl) column with core shell particles provided the selectivity and resolution to resolve warfarin and its 4-, 6-, 7-, and 10-hydroxy metabolites in addition to the internal standard naproxen in less than 3 min. This is the fastest analytical assay for warfarin and its major metabolites reported to date, making it ideal for metabolic studies. The applicability of the method was demonstrated by monitoring the metabolism of S-warfarin in human and rat liver microsomes, and evaluating the inhibitory effect of piperine on metabolite formation. The results showed that piperine inhibited the formation of the major metabolite, 7-hydroxywarfarin, with half-maximal inhibitory concentration (IC_50_) 14.2 μM and 3.2 μM in human and rat liver microsomes, respectively. Furthermore, coagulation studies *in vitro* using rat plasma showed that piperine does not affect prothrombin time (PT) and activated partial thromboplastin time (aPTT). This study suggested that piperine may present a potential drug interaction with warfarin at the metabolism level, but has no direct effect on the activation of the extrinsic or intrinsic coagulation cascades. Further clinical investigation is therefore required, as piperine may increase the bioavailability of warfarin, thus increasing risk of serious adverse events in patients.

## Highlights

• Piperine inhibits warfarin metabolism in human and rat liver microsomes.
• Fastest assay reported to detect warfarin and its major metabolites (<3 min).
• Piperine exhibits no impact on coagulation times (PT, aPTT) *in vitro* in rat plasma.
• Piperine may increase warfarin bioavailability, demanding caution in patient care.

## 1 Introduction

**Fig. 1:**
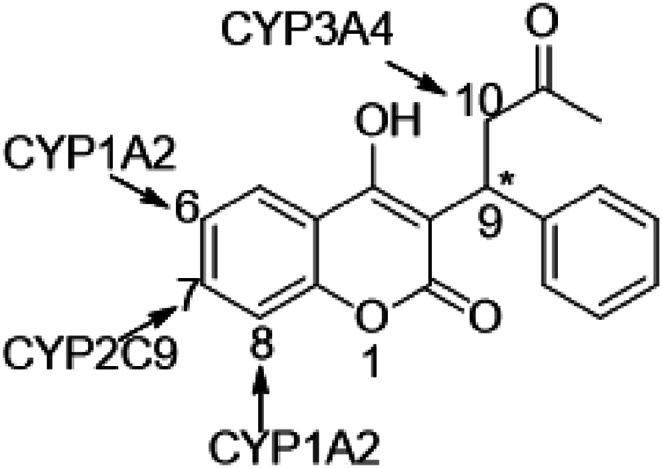
Warfarin structure and its sites of hydroxylation by Cytochrome P450 enzymes.

Warfarin is an anticoagulant frequently prescribed to individuals with atrial fibrillation, valvular heart disease, or a prosthetic heart valve to treat or prevent deep vein thrombosis, pulmonary embolism, and stroke [1]. Warfarin is a racemic mixture of S and R enantiomers with asymmetric carbon at position 9. The S-enantiomer has a 2–5 fold higher anticoagulant potency than the R-enantiomer [2]. S and R enantiomers also differ in their metabolism as they are stereoselectively metabolized by the hepatic Cytochrome P450 (CYP450) enzymes [3]. While R-warfarin is metabolized by CYP1A1, 1A2, and 3A4 to produce 6-, 8-, and 10-hydroxylated metabolites, S-warfarin metabolism by CYP2C9 produces 6- and 7-hydroxylated metabolites [4] (Fig. 1). Overall, CYP2C9 and CYP3A4 are the major CYP450 enzymes responsible for warfarin metabolism by the liver in human [5]. A change in the pharmacokinetics (PK) of warfarin after the administration of CYP450 inhibitors or inducers can enhance or diminish the anticoagulant activity and change the warfarin plasma concentration in the body [6].

Dietary supplement use and consumption of botanical products is increasing worldwide. Phytotherapy has been included in traditional medical systems, frequently serving as the primary means of healthcare in low- and middle-income nations [7]. The increase in medicinal plants use thus increases the likelihood of adverse effects and herb-drug interactions including pharmacokinetic alterations due to changes in the activity of CYP450 [8]. More importantly, the availability of the bioactive compounds in the pure form as a single agent or added with other bioactive compounds in dietary supplements may increase the potential of drug interactions due to the expected high concentrations of these compounds. Therefore, the use of isolated bioactive compounds as opposed to whole herb extract is commonly employed in CYP450 metabolism inhibition studies as it offers a simpler model and allows standardisation of the experimental conditions [[9], [10], [11]].

**Fig. 2:**
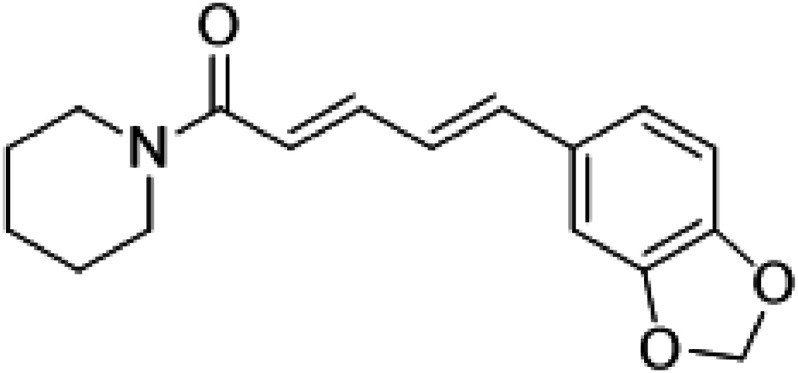
Structure of piperine.

Black and long pepper are among the herbal products and dietary supplements that have been shown to provide a variety of beneficial advantages for health. They have been found to possess several clinical properties such as anticoagulation, antihypertensive, antioxidant, anti-obesity and antihyperglycemic activities. These pharmacological properties are linked to the bioactive constituent piperine which is the principal isolated alkaloid of black pepper and long pepper [12,13] (Fig. 2).

Despite its beneficial effect, piperine has been reported to be a potent inhibitor of the metabolizing CYP450 enzymes, especially CYP3A4 [14], CYP2C9 [15], which are mainly responsible for warfarin metabolism.

Several methods for quantifying warfarin and its main metabolites in biological samples using high-performance liquid chromatography (HPLC) coupled with a variety of detectors have been reported including liquid chromatography tandem mass spectrometry (LC-MS/MS) [16], HPLC with ultraviolet detection (HPLC-UV) [17] and HPLC with fluorescence detection (HPLC-FLD) [18]. However, the reported methods either do not resolve the major warfarin metabolites or have a long run time. An ideal method for conducting metabolic studies would provide the selectivity to resolve the structurally related warfarin metabolites with short runtime.

Piperine was shown to alter the pharmacokinetics of warfarin in rats, however, the exact role of CYP metabolism in this interaction is not known yet. Therefore, this study aims to investigate the effect of piperine on warfarin CYP-mediated metabolism *in vitro* using human and rat liver microsomes. For this purpose, a new method using HPLC-FLD with PFP column was developed and applied in this study. In addition, the anticoagulation effect of piperine using rat plasma was investigated *in vitro* by conducting prothrombin time (PT) and activated partial thromboplastin time (aPTT) tests that measure the efficacy of the extrinsic and intrinsic coagulation system, respectively. The collective results of this study should provide insight to pharmacologists and clinicians on potential mechanistic interaction between piperine and warfarin and thus provide recommendation to patients on warfarin treatment.

## 2 Materials and method

### 2.1 Chemicals and reagents

Racemic warfarin, 7-hydroxywarfarin, 10-hydroxywarfarin, ketoconazole, sulfaphenazole, and piperine were purchased from Sigma Aldrich (St. Louis MO, USA). 4-hydroxywarfarin, and 6-hydroxywarfarin were acquired from Toronto Research Chemicals (Canada). S-warfarin (purity ≥98 %) were purchased from Cayman chemicals (USA). Acetonitrile (HPLC grade), methanol (HPLC grade), and ammonium acetate (HPLC grade) were requested from Fisher Scientific (USA). For microsomes incubation, human and rat liver microsomes, and 0.5 M phosphate buffer solution were purchased from Corning (USA). Nuclease-free water was purchased from Integrated DNA Technologies IDT company (USA). Nicotinamide adenine dinucleotide phosphate (NADPH) salt was purchased from Cayman chemicals (USA). To evaluate the activity of the microsomes, testosterone was purchased from Thermo Fisher Scientific (USA). Prothrombin time (PT) reagent, activated partial thromboplastin time (aPTT) reagent, and calcium chloride (0.02 M) were acquired from Biomed Diagnostics (USA). Naproxen (as an internal standard) and heparin were kindly supplied as gifts from Hikma Pharmaceuticals (Amman, Jordan).

### 2.2 Equipment

HPLC used was Shimadzu LC-2040C 3D plus HPLC system (Japan), which was equipped with a fluorescence detector (FLD), RF-20A. For warfarin, its metabolites, and the internal standard naproxen, the FLD was set at excitation and emission wavelengths; 310 nm and 390 nm, respectively. Data processing was performed on LabSolutions software version 6.87. Separation was achieved using Kinetex® pentafluorophenyl (PFP) column (100 × 3.0, 2.6 μm) with a mobile phase mixture consisting of 15 mM ammonium acetate buffer at pH 7 and methanol in a ratio of 60:40 (v/v) and delivered in isocratic mode at a flow rate of 0.7 mL/min. The chromatographic conditions are shown in Table 1.

**Table 1:** Optimized parameters of the proposed HPLC method.

Column	Kinetex® PFP column (100 × 3.0 mm, 2.6 μm)
Flow rate	0.7 mL/min
Injection volume	5 μL
Mobile phase and flow rate	60:40 v/v (15 mM ammonium acetate buffer pH 7: methanol)
Temperature	40 °C
Wavelengths (λ _ex_, λ _em_)	310, 390 nm

### 2.3 Preparation of standard solution

A stock solution of each of S-warfarin, 4, 6, 7, 10-hydroxywarfarin, and internal standard naproxen was prepared by dissolving a weight of 1 mg of reference standards in a 5 mL volumetric flask of methanol to produce a concentration of 200 μg/mL. Aworking solution was prepared by serial dilution using methanol to produce a 500 ng/mL concentration.

### 2.4 Sample preparation

#### 2.4.1 Microsomes incubation

The microsomal mixture consisted of human or rat liver microsomes (0.5 mg/mL), 100 mM phosphate buffer, and nuclease-free water was spiked with 2.6 μM S-warfarin. The mixture was pre-incubated at 37 °C for 5 min, then the reaction was started by adding 60 μL of NADPH (final concentration is 1 mM). Aliquots (100 μL) of the incubation solution were withdrawn at corresponding time points and added to 100 μL of ice-cold methanol (containing the internal standard, 1 μg/mL naproxen) to stop the reaction. The mixture was centrifuged at 4 °C, 10,000 rpm for 5 min, then 100 μL of the supernatant was transferred to HPLC for analysis.

### 2.5 HPLC method development

Different chromatographic conditions were experimented during the method development phase employing various types of mobile phases and stationary phases to separate S-warfarin and its metabolites. Initial experiments were done using Fortis diphenyl column (150 × 4.6 mm; particle size 3 μm) and a mobile phase of acetonitrile, methanol, and phosphate buffer 15 mM (pH 7) in a ratio of 10:20:70 at a flow rate of 1 mL/min. Several methods were then experimented by employing Kinetex® PFP column (150 × 4.6 mm; particle size 3 μm) and different compositions of mobile phase using various buffers and solvents including phosphate buffer 15 mM (pH 7), ammonium acetate buffer (15 mM, pH 7), methanol, and acetonitrile. Subsequent method development experiments employed Luna® PFP column (50 × 4.6 mm; particle size 5 μm) and a mobile phase of ammonium acetate buffer (15 mM, pH 7), and methanol in a ratio of 60:40 (v/v) at a flow rate of 1 mL/min. Final method was obtained using Kinetex® PFP column (100 × 3 mm; particle size 2.6 μm) and a mobile phase of ammonium acetate buffer (15 mM, pH 7), and methanol in a ratio of 60:40 (v/v) at a flow rate of 0.7 mL/min. Naproxen was used as an internal standard.

### 2.6 Effect of piperine on the metabolism of warfarin

#### 2.6.1 Effect of piperine on warfarin metabolism in human liver microsomes

The effect of piperine on warfarin metabolism was investigated in human liver microsomes by monitoring the formation of the major metabolites for 70 min using 5 concentrations (0, 10, 20, 50, 100 and 250 μM) of piperine. Ketoconazole as CYP3A4 inhibitor (1 μM) or sulfaphenazole as CYP2C9 inhibitor (2.5 μM) were used as positive controls. Incubation was performed as described above using 2.6 μM warfarin and sampling times at 0, 10, 20, 30, 40, 50, 60 and 70 min.

#### 2.6.2 Effect of piperine on warfarin metabolism in rat liver microsomes

To study the effect of piperine on warfarin metabolism in rat liver microsomes the formation of the major metabolites was monitored for 70 min using 5 concentrations (0, 1, 10, 20, 50 and 100 μM) of piperine. Ketoconazole (1 μM) was used as positive control. Sulfaphenazole was not used as it does not affect any isoenzyme in rat liver microsomes [19]. The incubation was performed as described above with 2.6 μM warfarin and sampling times at 0, 10, 20, 30, 40, 50, 60 and 70 min.

### 2.7 Study of piperine coagulation properties *in vitro* using rat plasma (Pharmacodynamic interaction)

#### 2.7.1 Animals and plasma samples preparation

A Male Wistar rat weighing about 250 g was obtained from the Jordan University of Science and Technology (JUST) animal house. The rat was kept in a clean room at 25 °C with 12 h of light-dark cycle and 50 % relative humidity. Filtered tap water and standard animal diet were provided. All experimental procedures were carried out under the approval of the Institutional Animal Care and Use Committee (IACUC; 636/12/4/16) of Jordan University of Science and Technology. Blood was collected according to sampling guidance by NC3Rs after rat decapitation in tubes containing 3.8 % trisodium citrate in a polypropylene container (9 parts of blood to 1 part of trisodium citrate solution). Blood was immediately centrifuged at 6500 rpm for 10 min, and plasma was separated [20].

#### 2.7.2 Prothrombin time test

Normal citrated rat plasma (75 μL) was mixed with normal saline (25 μL), used as a negative control, heparin (1 IU/mL final concentration), used as a positive control, or different concentrations of piperine (10, 50, 100 and 250 μM final concentration). The mixture was incubated for 10 min at 37 °C. PT reagent (100 μL), pre-incubated for 10 min at 37 °C was then added. The time required for clot formation was measured by eye observation and each experiment was repeated in triplicate.

#### 2.7.3 Activated partial thromboplastin time (aPTT) test

Samples were treated as above except that aPTT reagent (75 μL), pre-incubated for 10 min at 37 °C, was added instead of PT reagent and clotting was induced by adding 0.025 mol/L CaCl_2_ (75 μL). The time required for clot formation was measured by eye observation and each experiment was repeated in triplicate.

### 2.8 Metabolism inhibition and coagulation data analysis

The percent of inhibition was estimated by calculating the ratio of the peak area of the metabolite in presence of an inhibitor (piperine, sulfaphenazole, or ketoconazole) to the peak area of the metabolite without an inhibitor (control) at the final time point (70 min) and multiplying by 100 % as in Equation (1):

(1) $$\%\;inhibtion=\frac{Peak\;area\;of\;the\;metabolite\;with\;inhibitor\;at\;70\;\min}{Peak\;area\;of\;the\;metabolite\;without\;inhibitor\;at\;70\;\min}*100\%$$

To determine the effect of piperine on warfarin metabolism in liver microsomes, half-maximal inhibitory concentration (IC_50_) of piperine in human or rat liver microsomes was calculated by GraphPad Prism (version 8) using nonlinear regression analysis of the inhibitory percentage versus the logarithm of piperine concentration. The effect of piperine on aPTT and PT were reported as mean ± SD. GraphPad Prism 8 software was used for the statistical analyses. One way (ANOVA) and the Dunnett's multiple range test were performed. A p-value less than 0.05 indicates a significant difference between the control and experimental groups. A p-value of less than 0.05 was considered to be statistically significant.

## 3 Result and discussion

### 3.1 Method development

In the method development phase, several columns and mobile phases with different compositions were experimented to resolve warfarin and its metabolites from interferences present in the microsomes matrix. The aim was to develop a highly sensitive and selective method with the shortest possible run time. In addition to S-warfarin and the internal standard naproxen, samples contained 4, 6, 7, and 10-hydroxywarfarin metabolites which are the typical metabolites of S-warfarin in rat and human liver microsomes.

Previous published work conducted in our lab, described the development of HPLC-FLD method for the simultaneous determination of warfarin and 7-hydroxywarfarin in addition to the internal standard [18]. In the current work, the same conditions were attempted by using Fortis diphenyl column and a mobile phase containing acetonitrile, methanol, and phosphate buffer 15 mM (pH 7) in a ratio of 10:20:70 (v/v/v). The selectivity and resolution of the method, however, were not enough to resolve all metabolites even though various mobile phase compositions were tried.

In an attempt to enhance the selectivity and improve the separation of the methods, the chemistry of the stationary phase was changed to PFP. The PFP stationary phase has been recognized to have unique selectivity and therefore is commonly used as an alternative to common C_18_ and C_8_ alkyl stationary phases. It exhibits enhanced selectivity for the separation of positional isomers and non-planar molecules thus making it very relevant to the analysis of hydroxywarfarin metabolite isomers [21].

**Fig. 3:**
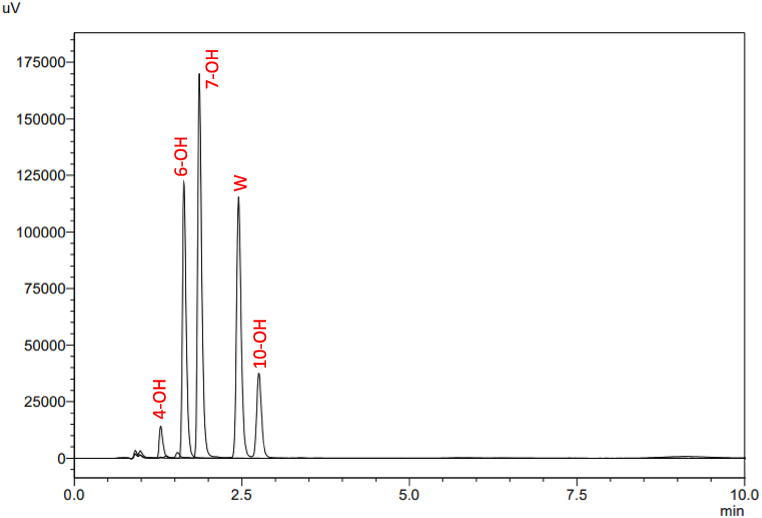
Representative HPLC chromatogram for warfarin and its metabolites (0.5 μg mL^−1^). Kinetex*®* PFP (100 × 3 mm; particle size 2.6 μm) a mobile phase of ammonium acetate buffer (15 mM, pH 7), and methanol in a ratio of 60:40 (v/v) at a flow rate of 0.7 ml/min.

The first PFP column experimented was Phenomenex Luna® PFP [22] which had the dimensions (50 × 4.6 mm; particle size 5 μm). Although less retention times and thus shorter runs could be achieved, all hydroxywarfarin metabolites could not be completely resolved, particularly warfarin and 10-OH metabolite, regardless of the mobile phase conditions. Therefore, in addition to changing the chemistry of the stationary phase, core-shell particle technology was introduced to improve the resolution of the method. A new PFP column was thus used: Kinetex® PFP column (150 × 4.6 mm; particle size 3 μm). Core-shell particles consist of a solid core covered with a porous layer that is applied either in layers or a single coating, depending on the manufacturer. Using core-shell particles in the column has the benefit of decreasing the volume of the pore space thus reducing the volume present for broadening from longitudinal diffusion (B term in the van Deemter equation). Also, due to the rapid mass transfer, the short diffusion path length can diminish the C term's contribution, thus improving the efficiency and shortening the analysis time [23]. The use of PFP with particle shell technology has significantly improved the separation for most of the analytes. However, in order to achieve the shortest run time possible, similar column was used with smaller particle size (Kinetex® PFP column 100 × 3 mm; particle size 2.6 μm). Fig. 3 shows the final optimized method with full resolution of warfarin, 4, 6, 7 and 10-hydroxywarfarin metabolites where all compounds eluted in less than 3 min. It is worth to mention that one of the strategies that adopted during the method development was to use chromatographic conditions that are compatible with MS detection by using only volatile buffers thus making the method transferable to LC-MS in future studies.

It should be noted that the primary goal of the study was to compare the percentage of relative peak area increase at the beginning and at the end of the incubation (0 and 70 min) as an indicator of the metabolism and clearance. Unlike typical HPLC applications, quantifying absolute drug or metabolite concentrations was not required in our study, eliminating the need for known reference standard concentrations and calibration curves.

### 3.2 Effect of piperine on the metabolism of warfarin

#### 3.2.1 Measurement of microsomes activity

To study the activity of the microsomes, testosterone (CYP 450 substrate) was incubated with the microsomes to confirm the validity of the microsomes [24]. Testosterone depleted quickly with no peak detectable after 10 min compared to similar incubations with No NADPH control (data not shown) indicating the microsomes were active and can be used to study warfarin metabolism.

#### 3.2.2 Effect of piperine on warfarin metabolism using human liver microsomes

**Fig. 4:**
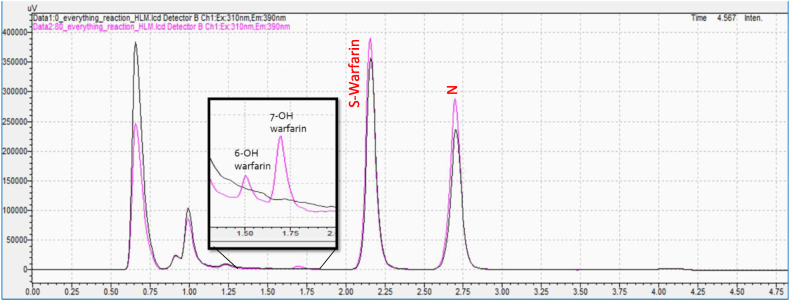
Representative HPLC chromatograms for metabolite formation incubation of S-warfarin in human liver microsomes at 0 min (black) and 70 min (pink) incubation time. Naproxen (N) was used as an internal standard. (For interpretation of the references to colour in this figure legend, the reader is referred to the Web version of this article.)

The metabolism of S-warfarin was selected to be studied since it is the pharmacologically active enantiomer of warfarin [25]. Also, its major route of metabolism is through CYP2C9 which yields 7-hydroxywarfarin, the main product of warfarin metabolism *in vitro* and in vivo [26,27]. S-warfarin was incubated, in duplicate, in a mixture of human liver microsomes, phosphate buffer, and nuclease-free water. The incubation was started by adding the cofactor NADPH. The result showed that 6-hydroxywarfarin and 7-hydroxywarfarin metabolites were formed, and their peak areas were increasing with time. These observations are in agreement with previous studies which showed that in human liver microsomes more than 80 % of the S-warfarin is converted to the two metabolites, 7-hydroxywarfarin and 6-hydroxywarfarin and formed in around a 3:1 ratio, respectively [28]. Fig. 4 shows 7-hydroxywarfarin and 6-hydroxywarfarin metabolite formation after 70 min with typical 3:1 peak ratio. These results demonstrate the sensitivity of the method and its suitability to monitor warfarin metabolite formation *in vitro*. In addition, Fig. 4 demonstrates the selectivity of the method as no matrix peaks can be observed at the corresponding retention times of the metabolites at the beginning of the incubation (0 min timepoint). This was also confirmed in all incubations with no NADPH cofactor (no metabolism).

**Fig. 5:**
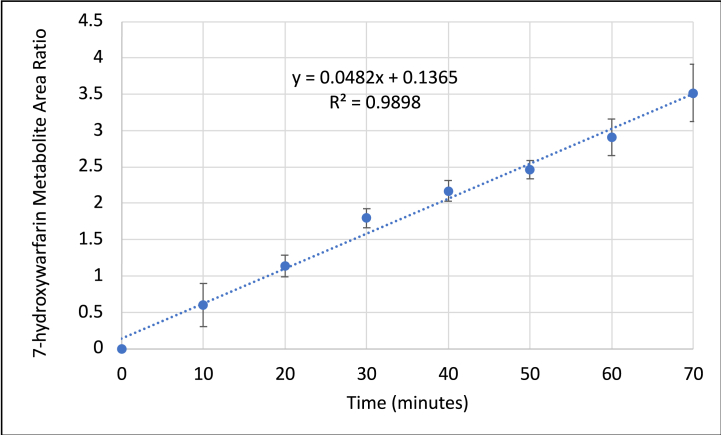
The linear formation of 7-hydroxywarfarin metabolite when incubated with human liver microsomes for 70 min. The fitted regression line is shown with the linear equation and coefficient of determination (R^2^). Data are shown as mean ± SD. n = 2.

The major metabolite 7-hydroxywarfarin, which has the highest metabolite peak area, was used as a marker for the metabolism of S-warfarin [[29], [30], [31]]. 7-hydroxywarfarin metabolite formation showed a linear rate in human liver microsomes up to 70 min incubation period (Fig. 5), therefore, peak area at 70 min was used for estimating the effect of inhibition. The linear rate of metabolite formation typically obtained in microsomes model confirms the validity of the method experimental setup and its reliability in producing inhibition data.

**Fig. 6:**
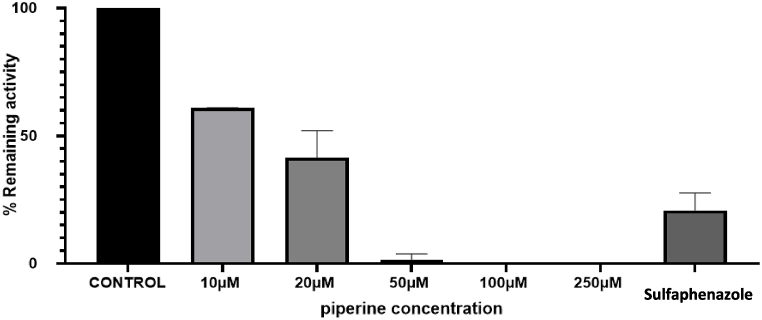
The effect of different concentrations of piperine (0, 10, 20, 50, 100, 250 μM) and 2.5 μM sulfaphenazole (as a positive control) on the formation of 7-hydroxywarfarin after incubation with human liver microsomes for 70 min. Data are shown as mean ± SD. n = 2.

The incubation experiments of S-warfarin were conducted in the presence of different concentrations of piperine (10, 20, 50, 100 and 250 μM) or inhibitors that were used as positive controls. The inhibitors used were ketoconazole (1 μM) and sulfaphenazole (2.5 μM) and the incubation was conducted for 70 min. The inhibition experiment results showed that ketoconazole did not affect the metabolism of S-warfarin as 7-hydroxywarfarin metabolite formation with and without ketoconazole were similar. This was expected since ketoconazole is a selective inhibitor for human CYP3A4 which is not involved in the metabolism of S-warfarin [32]. On the other hand, sulfaphenazole, a CYP2C9 positive inhibitor, decreased the metabolite formation of 7-hydroxywarfarin which is expected since this metabolite is a product of the CYP2C9 pathway in human [33]. Table 2 lists the percent of inhibitions of the different concentrations of piperine based on the decrease in 7-hydroxywarfarin metabolite formation. Fig. 6 shows metabolite formation in different piperine concentrations compared to sulfaphenazole. The results show that % of inhibition is increased with increasing piperine concentrations. Piperine completely inhibited metabolite formation at concentrations of 50 μM and higher with inhibition percentage 39 ± 0.1 % and 58 ± 7 % at 10 and 20 μM, respectively.

**Table 2:** The inhibitory effect of different concentrations of piperine on 7-hydroxywarfarin formation in human liver microsomes. Data are shown as mean ± SD. n = 2.

Piperine Concentration (μM)	% Inhibition
10	39 ± 0.1
20	59 ± 7
50	98 ± 1
100	99.9 ± 0.01
250	100

The results for human liver microsomes showing the percentage of inhibition for each tested concentration (Table 2) was calculated using Equation (1). The IC_50_ value (concentration producing a 50 % reduction in the control activity) for piperine was determined through nonlinear regression analysis by plotting the logarithm of the inhibitor concentration against the percentage of inhibition. IC_50_ value was calculated as 14.17 ± 1.1 μM obtained by GraphPad Prism (version 8) software.

Results shown in Table 2 and represented in Fig. 6 demonstrate their reproducibility and reliability. The studies were repeated in two independent experiments on different days at six different piperine concentrations showing excellent reproducibility. In addition, the integrity of the whole experimental procedure, including the HPLC component, was confirmed by the results of the positive control incubations (ketoconazole and sulfaphenazole) as discussed above demonstrating the fitness for the purpose of assessing metabolite inhibition in liver microsomes. Validity of the assay was also confirmed by the negative controls (No NADPH cofactor) that were included in each experiment, which confirmed that metabolite peak formation occurred only due to metabolism, ruling out other potential interfering factors like substrate dissociation/stability issues.

#### 3.2.3 Effect of piperine on warfarin metabolism using rat liver microsomes

**Fig. 7:**
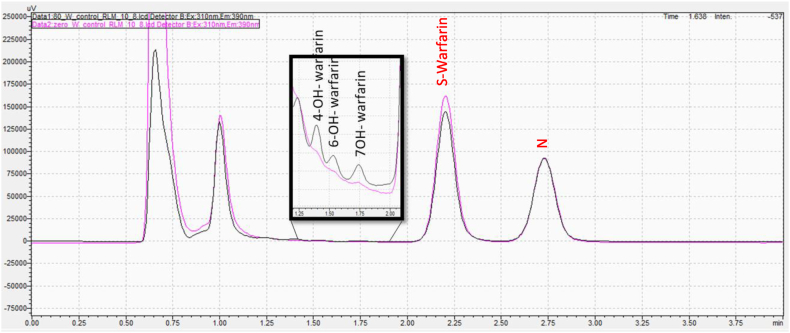
Representative HPLC chromatogram for metabolite formation after incubation of S-warfarin in rat liver microsomes for 70 min. N: Naproxen was used as an internal standard.

The same experiments performed in human liver microsomes were repeated in rat liver microsomes. Incubations of S-warfarin, however, did not produce the same metabolic profile as that in human liver microsomes. Here the metabolites formed were 4-OH, 6-OH, and 7-hydroxywarfarin metabolites (Fig. 7) compared to only 6 and 7-hydroxywarfarin metabolites in human liver microsomes. The formation of the metabolites was increasing over time. The formation of these three hydroxy metabolites were reported previously for the metabolism of S-warfarin in rat liver microsomes [33]. This is because the enzymes responsible for the metabolism of S-warfarin in rat are CYP2C6 and 2C11 unlike that in human where CYP2C9 is the major metabolizing enzyme of S-warfarin [34].

**Fig. 8:**
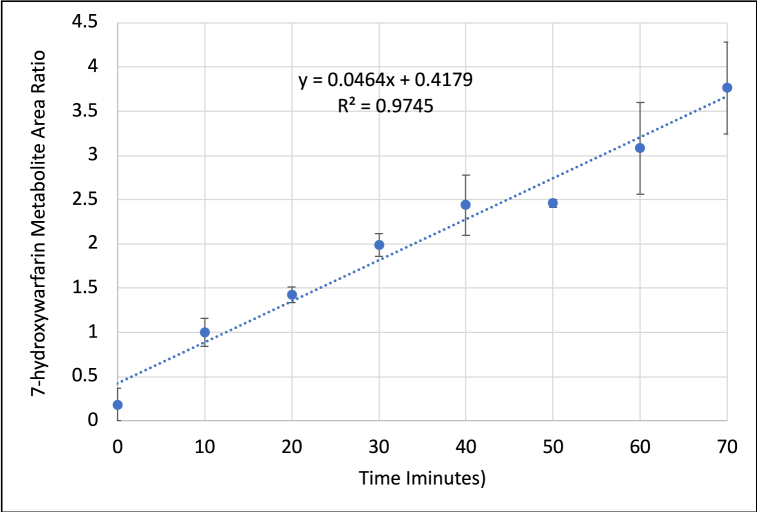
The linear formation of 7-hydroxywarfarin metabolite when incubated with rat liver microsomes for 70 min. The fitted regression line is shown with the linear equation and coefficient of determination (R^2^). Data are shown as mean ± SD. n = 2.

As in human liver microsomes, the major metabolite formed was 7-hydroxywarfarin, therefore, it was selected to monitor the rate of metabolism and study the effect of the inhibitors. 7-hydroxywarfarin metabolite formation showed a linear rate in rat liver microsomes throughout the 0–70 min incubation period (Fig. 8).

Incubations were conducted for S-warfarin in the presence of ketoconazole (1 μM), sulfaphenazole (2.5 μM), or piperine (1, 10, 20, 50 and 100 μM).

Results showed that sulfaphenazole did not inhibit the metabolism of S-warfarin in rat liver microsomes incubation as it did in human liver microsomes. This is because sulfaphenazole is a potent and selective inhibitor of human CYP2C9 [35] but not in rats [19]. Ketoconazole, on the other hand, caused a significant inhibition in 7-hydroxywarfarin metabolite formation in rats which was expected due to the low selectivity of ketoconazole toward CYP isoforms in rat thus its ability to inhibit all CYP isoforms [19]. In human, ketoconazole has no effect on 7-hydroxywarfarin metabolite formation as its affinity for CYP3A4 is more than 10-fold higher than that for the CYP2C9 responsible for the metabolism of S-warfarin and 7-hydroxywarfarin metabolite formation.

**Fig. 9:**
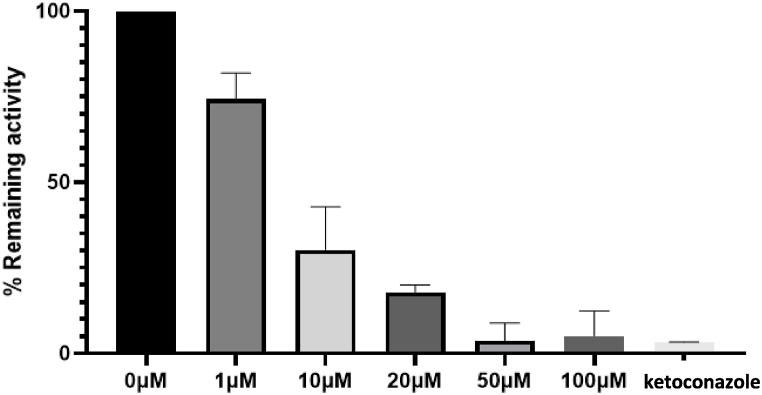
The effect of different concentrations of piperine (0, 1, 10, 20, 50, 100 μM) and 1 μM ketoconazole (as a positive control) on the formation of 7-hydroxywarfarin after incubation with rat liver microsomes for 70 min. Data are shown as mean ± SD. n = 2.

Studying the effect of piperine showed that increasing piperine concentration resulted in increased inhibition in 7-hydroxywarfarin metabolite formation (Table 3). The inhibitory effect of piperine can be observed in Fig. 9. The results showed that the piperine concentration of 50 μM and higher have almost complete inhibition of metabolite formation and was comparable to ketoconazole (positive control inhibitor). Inhibition was also observed at lower concentrations (1, 10 and 20 μM) indicating the potency of piperine in inhibiting 7-hydroxywarfarin metabolite formation.

**Table 3:** The inhibitory effect of different concentrations of piperine on 7-hydroxywarfarin formation in rat liver microsomes. Data are shown as mean ± SD. n = 2.

Piperine Concentration. (μM)	% Inhibition
1	26 ± 5
10	70 ± 9
20	82 ± 1
50	96 ± 4
100	95 ± 5

The results of this study complement that in our previous in vivo study in rats which showed that 7-hydroxywarfarin metabolite formation was decreased after co-administration of piperine. The results of the in vivo study, however, were not conclusive since warfarin concentrations were also decreased. Therefore the exact role of metabolism inhibition relative to other PK variables, such as oral bioavailability or protein binding, could not be determined [36]. In the current work the results using human and rat liver microsomes suggest that piperine inhibits warfarin metabolism which could be a major reason for altering the PK of warfarin in vivo [36]. The potency of piperine as an inhibitor for 7-hyrdoxywarfarin metabolite formation is demonstrated by the low value of half-maximal inhibitory concentration (IC_50_ = 3.24 ± 0.8 μM) as calculated in this study. It should be noted, however, that PK and PD are more complex in vivo as many variabilities and potential factors are involved such as absorption, drug transporters, protein binding and different metabolic pathways (e.g CYP450 3A, 9C) as we discussed in more details in our previous in vivo study [36].

### 3.3 Study of piperine coagulation properties using rat plasma

In our previous in vivo study in rats we showed that piperine unexpectedly lowered the concentration of warfarin and consequently produced lower INR values compared to warfarin only group [36]. Therefore, it was aimed in this current study to investigate if piperine has any effects on coagulation through activation of the extrinsic or intrinsic pathways which can be observed by conducting PT and aPTT testing, respectively. Since warfarin mechanism involves inhibiting synthesis of clotting factors, its use *in vitro* does not produce anticoagulation effect in the PT or aPTT tests, so its use is only valid for studies in vivo. Heparin was therefore used as a positive control for comparison purposes.

#### 3.3.1 Prothrombin time

**Fig. 10:**
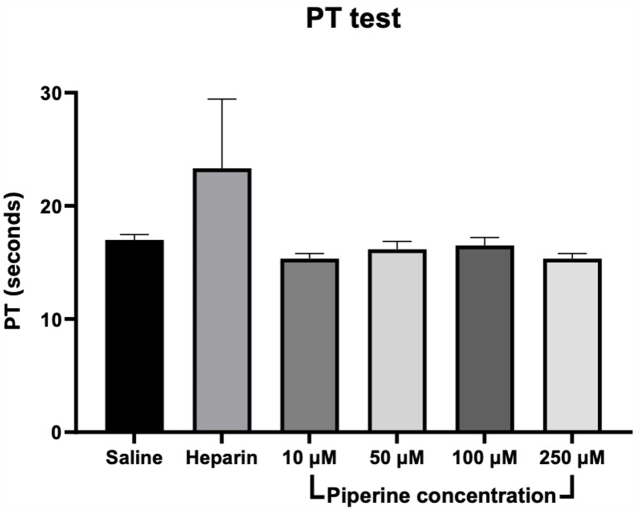
The effect of piperine (10, 50, 100 and 250 μM) on the prothrombin time test.

Prothrombin time is a test that measures the efficacy of the extrinsic coagulation. In this experiment, the prothrombin time was measured in rat plasma after adding different concentrations of piperine (10, 50, 100 and 250 μM) in comparison to normal saline (negative control), or heparin 1 IU/mL (positive control) (Fig. 10).

The results showed that piperine did not affect the prothrombin time even at high concentration (250 μM). This indicates that piperine has no direct effect on the activation of the extrinsic pathway of the coagulation cascade, suggesting no potential interaction with warfarin via this mechanism.

#### 3.3.2 Activated partial thromboplastin time (aPTT)

**Fig. 11:**
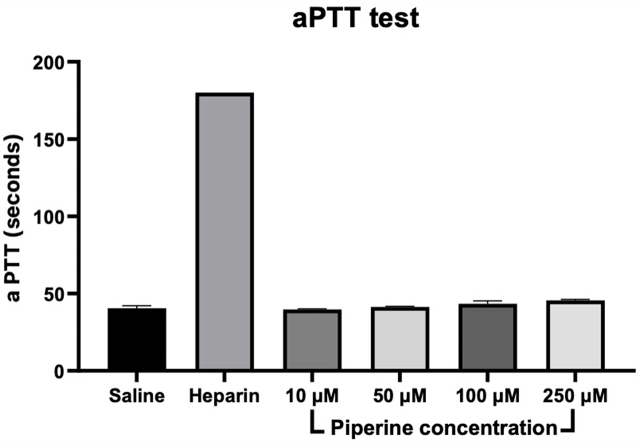
The effect of piperine (10, 50, 100, 250 μM) on the activated partial thromboplastin time test.

Activated partial thromboplastin time is a test that measures the efficacy of the intrinsic coagulation. Therefore the activated partial thromboplastin time was tested in rat plasma after adding several concentrations of piperine (10, 50, 100 and 250 μM) in comparison to normal saline (negative control), or heparin 1 IU/mL (positive control). The results shown in Fig. 11 indicate that piperine, compared to the negative control, had insignificant effect on aPTT. This indicates that piperine may not produce blood thinning properties through this mechanism. The positive control (heparin 1 IU/mL) has a significant effect on aPTT demonstrated as prolonging the time to coagulation for >180 s indicating the validity of the assay and results obtained.

## 4 Conclusion

This study investigated the effect of piperine on metabolism of warfarin using a rapid HPLC-Fluorescence method with PFP column demonstrating a fast and selective assay for monitoring major warfarin metabolites. Piperine exhibited inhibitory effects on the formation of the major 7-hydroxywarfarin metabolite, with IC_50_ values of 14.2 μM and 3.2 μM in human and rat liver microsomes, respectively. Coagulation studies in rat plasma indicated that piperine did not affect prothrombin time (PT) or activated partial thromboplastin time (APTT), suggesting it has no direct effect on extrinsic or intrinsic coagulation pathways.

This research indicates a potential interaction between piperine and warfarin at the metabolism level, demanding for further investigations due to the likelihood of increased warfarin bioavailability. The study not only provides a simple and economic analytical tool suitable for conducting metabolic studies, but also provides critical insights for pharmacologists and clinicians, emphasizing the need for cautious consideration of piperine-warfarin interactions in patients receiving warfarin treatment.

Further *in vitro* studies using human hepatocytes as well as clinical studies should be performed to establish that these results are relevant to patients treated in the clinic. Since the results suggested that the bioactive compound piperine inhibits warfarin metabolism, subsequent studies should focus on evaluating its effect in the herb extract form as in black pepper and long pepper extract to evaluate any effects from other ingredients that may cause synergy or reduction in the overall effect on warfarin and blood coagulation.

It should be noted that certain limitations exist in this study which can be addressed in the future. Patients on warfarin treatment receive racemic mixture of both S and R enantiomers, thus the effect of piperine should be evaluated on both forms even though that the S enantiomer is more potent. In addition, other metabolic pathways and metabolites can be monitored other than the major 7-hydroxywarfarin to have better understanding of the effect of piperine on warfarin metabolism. Moreover, the study does not account for potential factors that may affect warfarin metabolism, such as the genetic variations in CYP2C9 which is the major enzyme involved in S-warfarin metabolism. In addition, since performance characteristics of the method have not been estimated, full validation of the method should be considered so that the performance of the method is thoroughly evaluated particularly for quantifying samples at clinically relevant concentrations.

## Ethics statement

All animal experimental procedures were carried out under the approval of the Institutional Animal Care and Use Committee (IACUC; 636/12/4/16) of the Jordan University of Science and Technology, Irbid, Jordan.

## Data availability statement

Data will be made available on request.

## CRediT authorship contribution statement

**Aref L. Zayed:** Writing – review & editing, Writing – original draft, Supervision, Project administration, Methodology, Investigation, Funding acquisition, Conceptualization. **Mohammad Hadieh:** Writing – original draft, Investigation, Formal analysis, Data curation. **Jomana Al Hroot:** Writing – original draft, Methodology, Investigation, Formal analysis. **Fatima Hameedat:** Methodology, Formal analysis, Data curation. **Sana'a A. Jaber:** Writing – review & editing, Investigation, Data curation.

## Declaration of competing interest

The authors declare that they have no known competing financial interests or personal relationships that could have appeared to influence the work reported in this paper.
