# Supplementary material for: Development of a rapid HPLC-fluorescence method for monitoring warfarin metabolites formation: In vitro studies for evaluating the effect of piperine on warfarin metabolism and plasma coagulation
Source: Heliyon. 2024 May 14;10(10):e31266. doi: 10.1016/j.heliyon.2024.e31266 (PMC11130653; doi:10.1016/j.heliyon.2024.e31266)
Supplement: Multimedia component 1 [file mmc1.docx]

1. **Supplementary Figures**


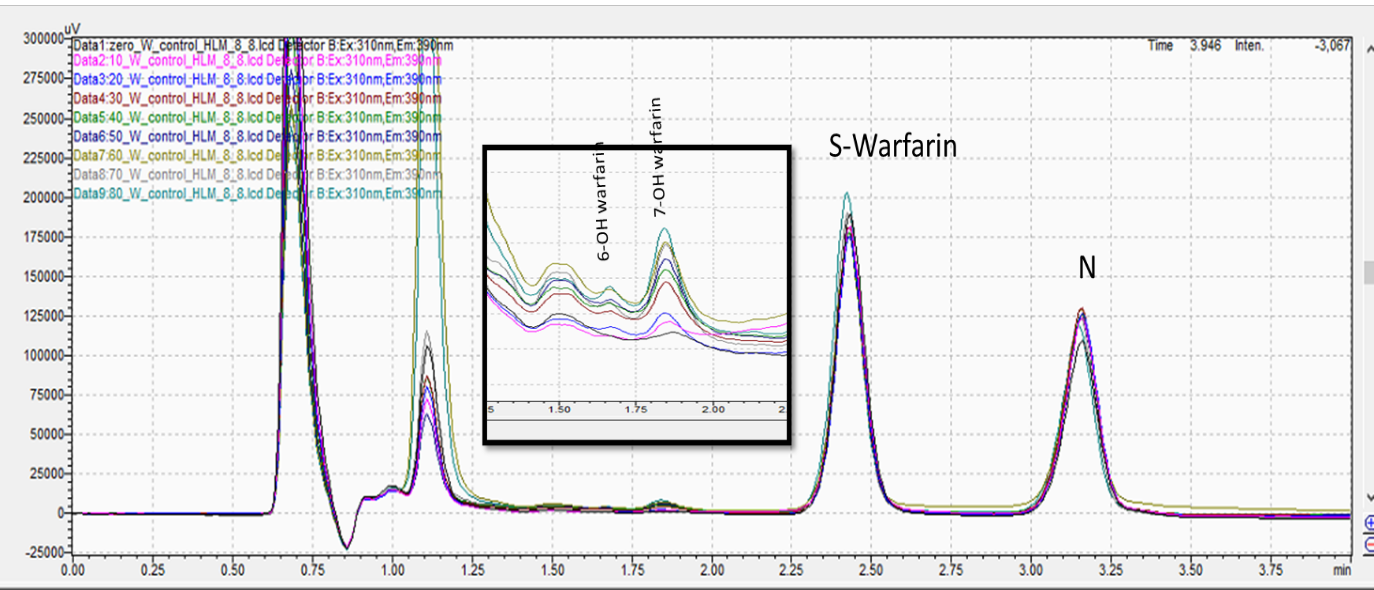


**Figure 1S:** Representative HPLC chromatograms for metabolite formation incubation of S-warfarin in human liver microsomes at all timepoints. Naproxen (N) was used as an internal standard


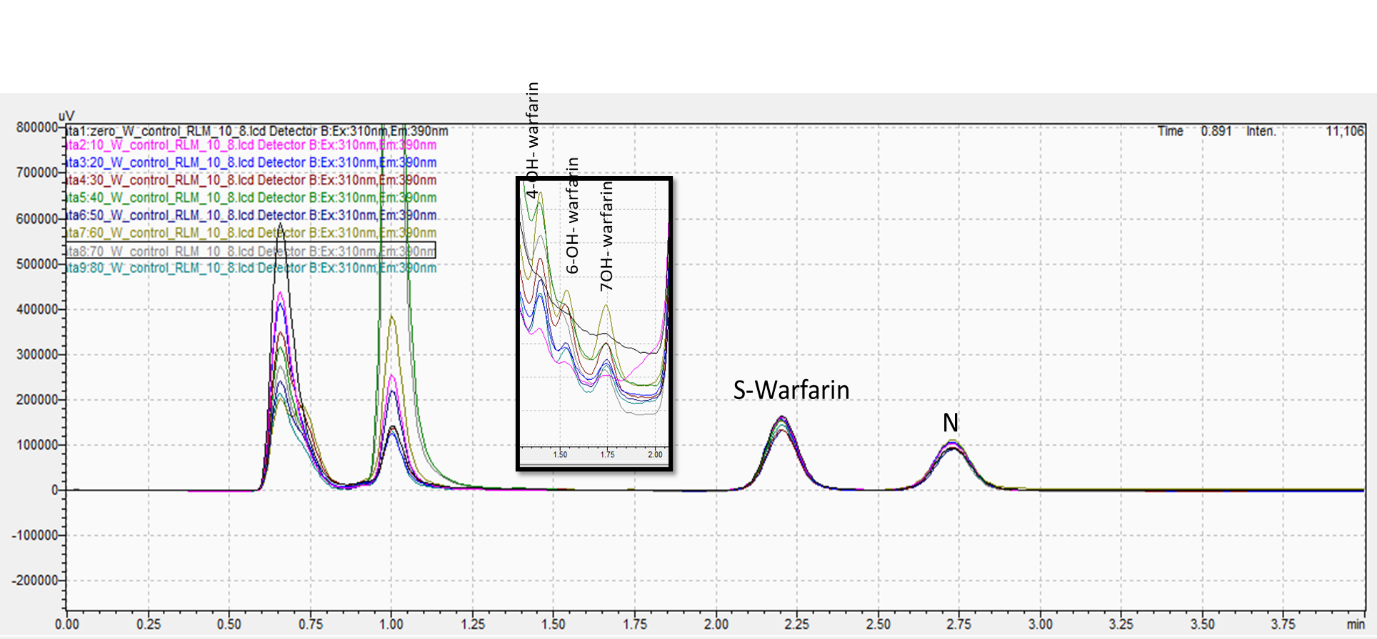


**Figure 2S:** Representative HPLC chromatograms for metabolite formation incubation of S-warfarin in rat liver microsomes at all timepoints. Naproxen (N) was used as an internal standard
